# Supplementary material for: Human Red Blood Cells as Oxygen Carriers to Improve Ex-Situ Liver Perfusion in a Rat Model
Source: J Clin Med. 2019 Nov 8;8(11):1918. doi: 10.3390/jcm8111918 (PMC6912657; doi:10.3390/jcm8111918)
Supplement: Supplementary file 1 [file jcm-08-01918-s001.pdf]

Supplementary Material

Table S1. *Brief review of ex-situ normothermic liver rat perfusion*

A search on PubMed, ISI Web of science, Springer, and Cochrane Library databases was performed. Only articles reporting data on machine perfusion systems in rats, with full text available were included. Conversely, we excluded all ex-situ perfusion systems without recirculating perfusion fluid. The time interval considered was from January 2005 to June 2019.

As machine perfusion is a promising field in preclinical research, a large number of papers was published over the last 15 years and 92 of them described liver MP in rodents. We reviewed all papers reporting rat ex-situ perfusions with recirculating fluid and 33 of them described NMP/NMRP (Supplementary 2),these results are the focus of this section.

A large discrepancy in NMP protocols was found and information was often incompletely reported. The duration of the NMP ranged from 20 to 360 min with a median perfusion time of 152 min (rewarming + normothermic phase). In 6 papers liver grafts were perfused through both portal vein and hepatic artery, while in other cases the graft was perfused only through the portal vein. A recent paper by the Groningen group(1) demonstrated comparable results between single (portal vein) and double (hepatic artery + portal vein) perfusion, thus reducing the technical issues related to HA perfusion. Pressure or flow controlled perfusion were independently adopted but these data are not generally described. While pressure-controlled perfusion protects endothelial cells, it can lead to incomplete liver perfusion. Conversely, flow-controlled perfusion could cause sinusoidal injury if flow resistances increase.

NMRP is based in almost all cases on the technique described by Bessems and colleagues(2) in which a single portal perfusion (3 mL/min/g liver weight) at 37°C with acellular perfusate is either preceded or not by a period of static rewarming to simulate the implantation time.

NMP circuit configuration in the present research includes characteristics derived from both the reviewed literature and our previous preclinical and clinical experience(3). A 150 min (30 min rewarming + 120 min evaluation) combined pressure (8 cmH_2O) and flow (2.5-3 mL/min/g liver weight) controlled single portal perfusion at 37°C is described here. The single portal perfusion was chosen for its simplicity and this choice was further supported by previous papers. Conversely, we used a combined pressure and flow controlled perfusion to avoid potential damage to sinusoids and hepatic architecture.

With regard to the time of perfusion, our choice was supported by the contribution of Imazis and colleagues(4). Indeed, they demonstrated a full metabolic recovery of ex-situ perfused grafts after 120 min.

Different Authors hypothesized that an optimized DO_2 during perfusion may result in a more rapid graft recovery after ischemia(4,5). As the partial pressure of oxygen and portal vein flow should not be raised to increase DO_2, we investigated the possibility to add an OxC to the perfusate. First, we calculated the predicted DO_2 (0.390 ml/min) and $\dot{V}O_2$ values without OxC. The calculated $\dot{V}O_2$ (0.330 ml/min) matched the $\dot{V}O_2$ values of other ex-situ series(6) and it was lower than in in-vivo experiments. The predicted values were confirmed by our DMEM group (DO_2 0.405 ± 0.053 ml/min and $\dot{V}O_2$ 0.325 ± 0.061) data. As we have already pointed out, in 1992 Mischinger et. al.(7) introduced the idea that ex-situ liver perfusion does not need high DO_2 and NMP set-up could be simplified by removing the OxC, namely erythrocytes. Among the reasons suggested to support the possibility to perform NMP with low oxygen delivery, a lower-than-physiological oxygen uptake ratio(8) and a sub-optimal take up of all the available oxygen (4,6) were demonstrated in some papers. The metabolic basis for these data was a damped metabolic activity rate during NMP, due to a low ex-situ metabolic activity, a mitochondrial inhibition due to hypoxia-induced factors and ischemia reperfusion injury may cause localized areas of hypoperfusion in the microvasculature. However, in most of these articles, perfusion fluid was not added with an OxC and some metabolic parameters demonstrated an incomplete reactivation of liver metabolism during ex-situ perfusion. For this reason we decided to increased DO_2 during perfusion to test our hypothesis that this strategy could protect rat liver grafts from hypoxic damage.

According to graft evaluation, bed-side and experimental markers were usually considered together to depict graft viability and in most of the cases laboratory markers were the aim of the papers. However, we were not able to find standardized biomarkers to asses graft quality. Transaminases were the most commonly used parameters to evaluate liver grafts during ex-situ normothermic perfusion. A comparison between different study group was usually performed and transaminase concentrations were directly related to the degree of parenchymal injury. However, we believe that this parameter has two main limitations: first we cannot determine what is the normal range of transaminases during NMP/NMRP and a direct comparison between groups could not highlight a possible damage in both groups. Second, liver graft injury, expressed by transaminase release, was rarely verified with an histological evaluation. Lactates level and trend are an easily measurable parameter that directly reflects liver metabolism in an isolated organ perfusion system. However,

less that 30% of the papers analyzed this biomarker. Bile production is a promising tool for liver viability, however no definitive data are reported and only a small number of information is available to support bile production as a viability marker(9).

Table S2. *Summary of the main characteristics of normothermic ex-situ perfusion protocols on rats published in literature*

Author	Year	Donor Type	Durata SCS (h)	MP type	PV/PV + HA	NMRP duration (min)	Perfusate volume (ml)	Blood	Ht	Blood origin	PV Flow (ml/min)	PV pressure (mmHg)	Blie production (ml/h)	Graft evaluation
Stadler M[10]	2005	DBD + DCD	/	NMP	PV	145	125	no	/	/	5	9	/	Lac
Bessems M[11]	2005	DBD	24	NMRP	PV	60	250	no	/	/	30	/	0.39	AST
Bessems M[12]	2005	DBD	0	NMRP	PV	60	/	no	/	/	/	/	0.2	AST
Xu H[13]	2005	DBD	24	NMRP	PV	30	/	no	/	/	/	/	0.49	H&E
Dutkowski P[5]	2006	DCD	5	NMRP	PV	180	50	yes	6.4	rat	16.5	6.6	0.17	Lac
Stadler M[14]	2007	DCD	/	NMP	PV	60	125	no	/	/	5	12	/	Lac
Bessems M[15]	2007	DBD	24	NMRP	PV	60	250	no		/	38	16	0.26	AST
Tolboom H[16]	2008	DCD	2	NMP	PV	240	60	yes	18	rat	/	/	0.50	AST
Vairetti M[17]	2008	DBD	6	NMP	PV	360	200	no	/	/	/	/	0.02	AST
Manekeller S[18]	2008	DCD	18	NMRP	PV	120	/	no	/	/	30	/	0.16	AST
Saad S[19]	2008	DCD	16	NMRP	PV	45	/	no	/	/	30	/	/	AST + Lac
Xu H[20]	2008	DBD	24	NMRP	PV	30	200	no	/	/	15	4	/	H&E
Stegemann J[21]	2009	DCD	18	NMRP	PV	120	250	no	/	/	30	4.5	/	AST + Lac
Olschewski P[22]	2010	DCD	6	NMRP	PV	60	/	no	/	/	30	/	0.28	AST
Perk S	2011	DCD	/	NMP	PV	/	60	yes	18	rat	n.d.	8	/	H&E
Orman M[23]	2011	DBD	0	NMP	PV+HA	60	500	yes	10	bovine	36	15	/	BUN + Chet
Perk S[6]	2012	DCD	/	NMP	PV	/	60	yes	18	rat	n.d.	8	/	H&E
Tolboom H[24]	2012	DCD	5	NMP	PV	300	60	yes	18	rat	20	9	0.70	AST
Izamis ML[4]	2013	DBD +DCD	/	NMP	PV	300	60	yes	18	rat	18	11	/	Lac
Carnevale ME[25]	2013	DCD	24	NMRP	PV	90	/	no	/	/	35	10.3	0.12	AST
Stegemann J[26]	2013	DCD	/	NMRP	PV	120	/	/	/	/	30	/	/	AST + Lac
Schlegel A[27]	2014	DBD + DCD	4	NMP	PV+HA	200	50	yes	15	rat	20	8	0.30	H&E
Niu X[28]	2014	DCD	/	NMRP	/	120	350	no	/	/	20	/	0.1	H&E
Tarantola E[29]	2014	DBD	6	NMRP	PV	120	200	no	/	/	/	/	/	H&E
Ferrigno A[30]	2015	DBD	/	NMP	PV	360	/	no	/	/	26	4	0.30	Lac
Westerkamp AC[31]	2015	DCD	6	NMRP	PV+HA	120	100	yes	25	human		11	/	H&E
op den Dries S[32]	2016	DBD +DCD	3	NMP	PV+HA	180	100	yes	17.5	human	25	11	0.60	AST
Berardo C[33]	2017	DBD	6	NMRP	PV	120	/	no	/	/	/	/	0.24	AST
Okamura Y[34]	2017	DBD	4	NMRP	PV+HA	120	150	no	/	/	30	4.7	0.23	AST
von Horn C[35]	2017	DCD	18	NMRP	PV	120	/	no	/	/	30	/	0.3	AST
Zeng C[36]	2017	DCD	3	NMRP	PV	120	150	no	/	/	17	10.3	0.09^	AST
Rigo F[37]	2018	DBD	na	NMP	PV	180	70	yes	9	rat	1.3*	9	0.04^	AST; Lac; Bile
Brüggenwirth IMA [1]	2018	DCD	6	NMRP	PV/PV+HA	120	110	yes	/	rat	/	11	0.25	AST; Lac; Bile
**Mean values**			**10 ± 9**			**140 ± 90**	**153 ±111**		**16 ± 5**		**24 ± 9**	**9 ±3**	**0.29 ± 0.17**	

Table S3. *Reagents used during experiments*

Reagent	Manufacturer
Thiopental sodium, 0.5 g	Inresa Arzneimittel GmbH, Freiburg, Germany
Heparin, 5000 UI/ml	I.B.N. Savio, Roma, Italy
Basic Glutaster	Farmec, Settimo di Pescantina, Italy
Albumin, 0.2 g/ml 20% immuno	Baxter S.p.A., Roma, Italy
NaCl, 0.9%	Baxter S.p.A.
Gas mixture of CO_2 (5%) and O_2 (95%)	Sapio S.r.l., Monza, Italy
Celsior solution	Groupe IGL, Lissieu, France
DMEM	Sigma-Aldrich, St.Louis, MO, USA
Human albumin	Kedrion, Biopharma, Lucca, Italy
Streptomycin - penicillin	Gibco, life Technologies
Glutamine	Gibco, life Technologies
Insulin	Humalog, Eli Lilly, Nederland B.V.

Table S4. *Instruments employed during experiment*

Reagent	Manufacturer
Rats	Envigo, Udine, Italy
Ventilated cage	Tecniplast S.p.A., Varese, Italy
Roller pump	Ismatec, Wertheim, Germany
Heat exchanger Ecoline E 103	Lauda Dr. R. Wobser Gmbh & Co. Kg, Lauda-Konigshofen, Germany
Automatic blood gas analyzer ABL 800 FLEX	A. De Mori Strumenti, Milano, Italy
Data acquisition software Colligo	Elekton, Milano, Italy
Oven	LTE Scientific, Greenfield, United Kingdom
Double-headed surgical microscope OPMI 1-	Zeiss West Germany, Oberkochen, Germany
1.3 mm temperature probe	Panlab Harvard Apparatus
Parafilm	Sigma-Aldrich, St. Louis, MO, USA
Circulating tube	Benefis S.R.L. Genova, Italy

Artificial lung	Micro-1 Rat Oxygenation, Dongguan Kewei, Medical Instrument, Dongguan City, China
Pressure column	TruWave, Edwards Lifesciences, Irvine, CA, USA
Glass reservoir	Hugo Sachs Elektronik-Harvard apparatus, March Germany
16 gauge cannula	Introcan- W Certo, Braun, Melsungen, Germany
ELISA kit for Hyaluronic Acid	R&D Systems, Minneapolis, MN, USA

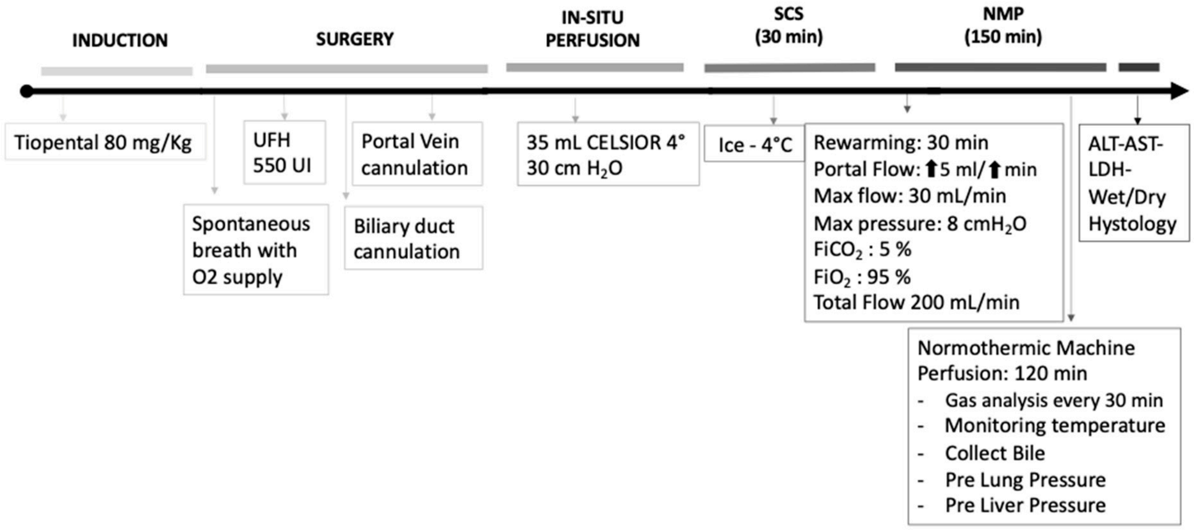

Figure S1. *Schematic representation of experimental workflow. After induction of anesthesia, biliary duct and portal vein were surgically cannulated, and liver was in situ perfused with cold Celsior solution (IGL, France). After 30 minutes of static cold storage, the liver was connected to perfusion machine and rewarmed until 37° C in 30 minutes. The normothermic perfusion at 37° lasted for 120 minutes. Every 30 minutes gas analysis were performed from post liver perfusate and every 60 minutes from both pre liver and post liver perfusate. Bile was collected, temperature, pre-lung and pre-liver pressures were monitored. At the end of the perfusion liver biopsies were collected*.

Table S5. *Characteristics of the perfusion fluid in the two experimental groups: concentration of drugs and components of the two perfusion fluids. * volume of red-blood packed cells to reach an estimated hematocrit of 15%. Stre-pen, streptomycin-penicillin*

	Volume (ml)	
	DMEM	BLOOD
**DMEM**	76	56
**Human Albumin 4%**	20	20
**pRBC**	0	20*
**Stre-pen (10 ug/ml-104 UI/mL)**	1	1
**Glutamine (200 mM/ml)**	1	1
**Actrapid (100 UI/mL)**	2	2
**Heparin (5000 UI/mL)**	0	0.1
**Total Volume**	**100**	**100.1**

$$[1,34 \text{ x Hb (g/dL)}_{\text{PRE-LIVER}} \text{ x HbO2(\%)}] + 0,003 \text{ x P}_{\text{PRE-LIVER}}O_2\text{(mmHg)} =$$

Content of Oxygen of the pre-liver perfusate ($C_{PRE}O_2$)

$$[1,34 \text{ x Hb (g/dL)}_{\text{POST-LIVER}} \text{ x HbO2(\%)}] + 0,003 \text{ x P}_{\text{POST-LIVER}}O_2\text{ (mmHg)} =$$

Content of Oxygen of the post-liver perfusate ($C_{POST}O_2$)

$$C_{PRE}O_2 - C_{POST}O_2 = \boldsymbol{\Delta_{PRE\text{-}POST}}$$

$$\Delta_{PRE\text{-}POST} \text{ x Pump flow (mL/min) x 10} = \boldsymbol{\dot{V}O_2}$$

In an analogous way, Oxygen delivery (DO_2) was measured as follows: $C_{PRE}O_2$ x Pump flow (mL/min) x 10

Figure S2. *Modified Fick equation used to calculate $\dot{V}O_2$ and DO_2 in our experiments. Cardiac output was intended as the pump flow; Pre-liver perfusate samples were intended as O2 enriched perfusate (arterial blood of the Fick equation), whereas post-liver perfusate samples, collected directly from the IVC, were used for the calculation of venous oxygen content in the Fick equation.*

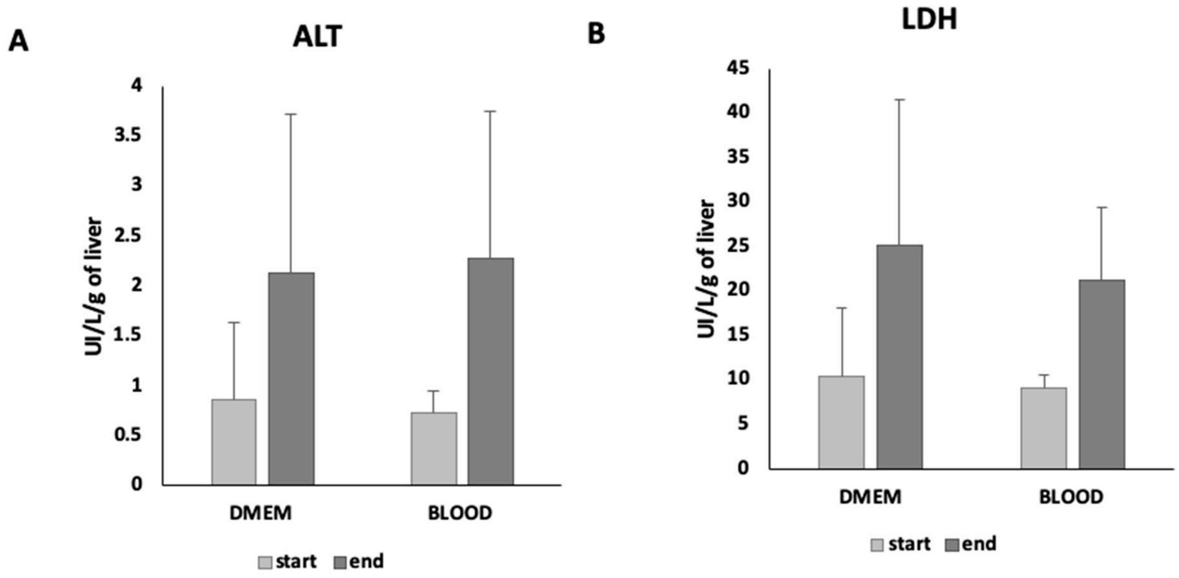

Figure S3. *Hepatocellular damage markers during normothermic machine perfusion. (**A**) postoperative AST levels and (**B**) lactates dehydrogenases (LDH) levels*

References:

1. Brüggenwirth IMA, Moore C, Mahboub P, et al. A Comparative Study of Single and Dual Perfusion During End-ischemic Subnormothermic Liver Machine Preservation Isabel. *Transplant Direct*. 2018;4:1-8. doi:10.1097/TXD.0000000000000840
2. Machine C, Using P, Bessems M, Doorschodt BM, Gulik TM Van. Improved Rat Liver Preservation by Hypothermic Enriched Preservation Solution. *Liver Transplant*. 2005;11(5):539-546. doi:10.1002/lt.20388
3. Dondossola D, Lonati C, Zanella A, et al. Preliminary experience on hypothermic oxygenated machine perfusion in an Italian Liver Transplant Center. *Transplant Proc*. 2018;ongoing pu. http://doi.wiley.com/10.1002/lt.25315.
4. Izamis ML, Tolboom H, Uygun B, Berthiaume F, Yarmush ML, Uygun K. Resuscitation of Ischemic Donor Livers with Normothermic Machine Perfusion: A Metabolic Flux Analysis of Treatment in Rats. *PLoS One*. 2013;8(7). doi:10.1371/journal.pone.0069758

5. Dutkowski P, Furrer K, Tian Y, Graf R, Clavien P-A. Novel Short-term Hypothermic Oxygenated Perfusion (HOPE) System Prevents Injury in Rat Liver Graft From Non-Heart Beating Donor. *Ann Surg*. 2006;244:968-977. doi:10.1097/01.sla.0000247056.85590.6b
6. Perk S, Izamis ML, Tolboom H, et al. A metabolic index of ischemic injury for Perfusion-Recovery of cadaveric rat livers. *PLoS One*. 2011;6(12):1-11. doi:10.1371/journal.pone.0028518
7. Mischinger HJ, Walsh TR, Liu T, et al. An Improved Technique for Isolated Perfusion of Rat Livers and an Evaluation of Perfusates. *J Surg Res*. 1992;53(2):158-165.
8. Imber CJ, St.Peter SD, Cenarruzabeitia DE, et al. Advantages of normothermic perfusion over cold storage in liver preservation. 2002;73(5):701-709.
9. Watson CJE, Jochmans I. From “Gut Feeling” to Objectivity: Machine Preservation of the Liver as a Tool to Assess Organ Viability. *Curr Transplant reports*. 2018;5(1):72-81. doi:10.1007/s40472-018-0178-9
10. Stadler M, Nuyens V, Seidel L, Albert A, Boogaerts JG. Effect of Nutritional Status on Oxidative Stress in an Ex Vivo Perfused Rat Liver. *Anesthesiology*. 2005;103:978-986.
11. Bessems M, Doorschodt BM, Marle J Van, Vreeling H, Meijer AJ, Gulik TM Van. Improved Machine Perfusion Preservation of the Non- Heart-Beating Donor Rat Liver Using Polysol : A New Machine Perfusion Preservation Solution. *Liver Transplant*. 2005;11(11):1379-1388. doi:10.1002/lt.20502
12. Bessems M, Doorschodt BM, Hooijschuur O, Vliet AK Van, Gulik TM Van. Optimization of a New Preservation Solution for Machine Perfusion of. 2004:329-331. doi:10.1016/j.transproceed.2004.12.220
13. Xu H, Zhang JX, Jones JW, Southard JH, Clemens MG, Lee CY. Hypothermic Machine Perfusion of Rat Livers Preserves Endothelial Cell Function. 2004:335-337. doi:10.1016/j.transproceed.2004.11.075
14. Stadler M, Nuyens V, Boogaerts JG. Intralipid minimizes hepatocytes injury after anoxia-reoxygenation in an ex vivo rat liver model 2007. *Nutrition*. 2007;23:53-61.
15. Bessems M, Doorschodt BM, Kolkert JLP, et al. Preservation of Steatotic Livers : A Comparison Between Cold Storage and Machine Perfusion Preservation. 2007:497-504. doi:10.1002/lt.
16. Tolboom H, Milwid JM, Izamis M-L, Uygun K, Berthiaume F, Yarmush ML. SEQUENTIAL COLD STORAGE AND NORMOTHERMIC PERFUSION OF THE ISCHEMIC RAT LIVER. *Transplant Proc*.

2008;40(5):1306-1309. doi:10.1016/j.transproceed.2008.03.100.SEQUENTIAL
17. Vairetti M, Ferrigno A, Rizzo V, et al. Correlation Between the Liver Temperature Employed During Machine Perfusion and Reperfusion Damage : Role of Ca 2+. *Liver Transplant*. 2008;14:494-503. doi:10.1002/lt.21421
18. Manekeller S, Schuppius A, Stegemann J, Hirner A, Minor T. Role of perfusion medium , oxygen and rheology for endoplasmic reticulum stress-induced cell death after hypothermic machine preservation of the liver. 2008;21:169-177. doi:10.1111/j.1432-2277.2007.00595.x
19. Saad S, Minor T. Short-Term Resuscitation of Predamaged Donor Livers by Brief Machine Perfusion: The Influence of Temperature. *TPS*. 2008;40(10):3321-3326. doi:10.1016/j.transproceed.2008.06.058
20. Xu H, Lee CY, Clemens MG, Zhang JX. Inhibition of TXA2 synthesis with OKY-046 improves liver preservation by prolonged hypothermic machine perfusion in rats. *J Gastroenterol Hepatol*. 2008;23(7 PT2). doi:10.1111/j.1440-1746.2007.05061.x
21. Stegemann J, Hirner A, Rauen U, Minor T. Cryobiology Gaseous oxygen persufflation or oxygenated machine perfusion with Custodiol-N for long-term preservation of ischemic rat livers ? q. *Cryobiology*. 2009;58(1):45-51. doi:10.1016/j.cryobiol.2008.10.127
22. Olschewski P, Gaß P, Ariyakhagorn V, et al. Cryobiology The influence of storage temperature during machine perfusion on preservation quality of marginal donor livers q. *Cryobiology*. 2010;60(3):337-343. doi:10.1016/j.cryobiol.2010.03.005
23. Orman MA, Ierapetritou MG, Androulakis IP, Berthiaume F. Metabolic Response of Perfused Livers to Various Oxygenation Conditions. 2011;108(12):2947-2957. doi:10.1002/bit.23261.Metabolic
24. Tolboom H, Izamis M-L, Sharma N, et al. Subnormothermic Machine Perfusion at Both 20°C and 30°C Recovers Ischemic Rat Livers for Successful Transplantation. 2012;175(1):1-17. doi:10.1016/j.jss.2011.03.003.Subnormothermic
25. Carnevale ME, Balaban CL, Guibert EE, Bottai H, Rodriguez J V. Hypothermic machine perfusion versus cold storage in the rescuing of livers from non-heart-beating donor rats. *Artif Organs*. 2013;37(11):985-991. doi:10.1111/aor.12235
26. Stegemann J, Hirner A, Rauen U, Minor T. Use of a New Modified HTK Solution for Machine Preservation of Marginal Liver Grafts. *J Surg Res*. 2010;160(1):155-162. doi:10.1016/j.jss.2008.10.021
27. Schlegel A, Kron P, Graf R, Dutkowski P, Clavien PA. Warm vs. cold perfusion techniques to

rescue rodent liver grafts. *J Hepatol*. 2014;61(6):1267-1275. doi:10.1016/j.jhep.2014.07.023
28. Niu X, Huang WH, Boer B De, Delriviere L. Iron-Induced Oxidative Rat Liver Injury After Non – Heart-Beating Warm Ischemia Is Mediated by Tumor Necrosis Factor a and Prevented by Deferoxamine. *Liver Transplant*. 2014;20:904-911. doi:10.1002/lt.23893
29. Tarantola E, Bertone V, Milanesi G, et al. Dipeptidylpeptidase-IV activity and expression reveal decreased damage to the intrahepatic biliary tree in fatty livers submitted to subnormothermic machine-perfusion respect to conventional cold storage. *Eur J Histochem*. 2014;58(3):2414. doi:10.4081/ejh.2014.2414
30. Ferrigno A, Giuseppina L, Pasqua D, et al. Metabolic shift in liver : Correlation between perfusion temperature and hypoxia inducible factor-1 α. *World J Gastroenterol*. 2015;21(4):1108-1116. doi:10.3748/wjg.v21.i4.1108
31. Westerkamp AC, Mahboub P, Meyer SL, et al. End-Ischemic Machine Perfusion Reduces Bile Duct Injury In Donation After Circulatory Death Rat Donor Livers Independent of the Machine Perfusion Temperature. 2015:1300-1311. doi:10.1002/lt.
32. op den Dries S, Karimian N, Westerkamp AC, et al. Normothermic machine perfusion reduces bile duct injury and improves biliary epithelial function in rat donor livers. *Liver Transplant*. 2016;22(7):994-1005. doi:10.1002/lt.24436
33. Berardo C, Giuseppina L, Pasqua D, Ferrigno A. Machine Perfusion at 20 ° C Prevents Ischemic Injury and Reduces Hypoxia-Inducible Factor-1 a Expression During Rat Liver Preservation. *Ann Transplant*. 2017;22:581-589. doi:10.12659/AOT.904631
34. Okamura Y, Hata K, Tanaka H, et al. Impact of Subnormothermic Machine Perfusion Preservation in Severely Steatotic Rat Livers: A Detailed Assessment in an Isolated Setting. *Am J Transplant*. 2016;(3):1204-1215. doi:10.1111/ajt.14110
35. Horn C Von, Baba HA, Hannaert P, et al. Controlled oxygenated rewarming up to normothermia for pretransplant reconditioning of liver grafts. *Clin Transplant*. 2017;31. doi:10.1111/ctr.13101
36. Zeng C, Hu X, He W, et al. Hypothermic machine perfusion ameliorates inflammation during ischemia-reperfusion injury via sirtuin-1-mediated deacetylation of nuclear factor-κB p65 in rat livers donated after circulatory death. *Mol Med Rep*. 2017;16(6):8649-8656. doi:10.3892/mmr.2017.7738
37. Rigo F, Stefano N De, Navarro-tableros V, et al. Extracellular Vesicles from Human Liver Stem Cells Reduce Injury in an Ex Vivo Normothermic Hypoxic Rat Liver Perfusion Model.

2018;102(5):205-210. doi:10.1097/TP.0000000000002123
